# Organs to Cells and Cells to Organoids: The Evolution of *in vitro* Central Nervous System Modelling

**DOI:** 10.3389/fncel.2019.00129

**Published:** 2019-04-09

**Authors:** Dario Pacitti, Riccardo Privolizzi, Bridget E. Bax

**Affiliations:** ^1^Molecular and Clinical Sciences Research Institute, St George’s, University of London, London, United Kingdom; ^2^College of Medicine and Health, St Luke’s Campus, University of Exeter, Exeter, United Kingdom; ^3^Gene Transfer Technology Group, Institute for Women’s Health, University College London, London, United Kingdom

**Keywords:** CNS, hiPSC, human neurons, human glia, neurogenesis, neurological disorders, cerebral organoids, organotypic

## Abstract

With 100 billion neurons and 100 trillion synapses, the human brain is not just the most complex organ in the human body, but has also been described as “the most complex thing in the universe.” The limited availability of human living brain tissue for the study of neurogenesis, neural processes and neurological disorders has resulted in more than a century-long strive from researchers worldwide to model the central nervous system (CNS) and dissect both its striking physiology and enigmatic pathophysiology. The invaluable knowledge gained with the use of animal models and *post mortem* human tissue remains limited to cross-species similarities and structural features, respectively. The advent of human induced pluripotent stem cell (hiPSC) and 3-D organoid technologies has revolutionised the approach to the study of human brain and CNS *in vitro*, presenting great potential for disease modelling and translational adoption in drug screening and regenerative medicine, also contributing beneficially to clinical research. We have surveyed more than 100 years of research in CNS modelling and provide in this review an historical excursus of its evolution, from early neural tissue explants and organotypic cultures, to 2-D patient-derived cell monolayers, to the latest development of 3-D cerebral organoids. We have generated a comprehensive summary of CNS modelling techniques and approaches, protocol refinements throughout the course of decades and developments in the study of specific neuropathologies. Current limitations and caveats such as clonal variation, developmental stage, validation of pluripotency and chromosomal stability, functional assessment, reproducibility, accuracy and scalability of these models are also discussed.

## Introduction

**FIGURE 1:**
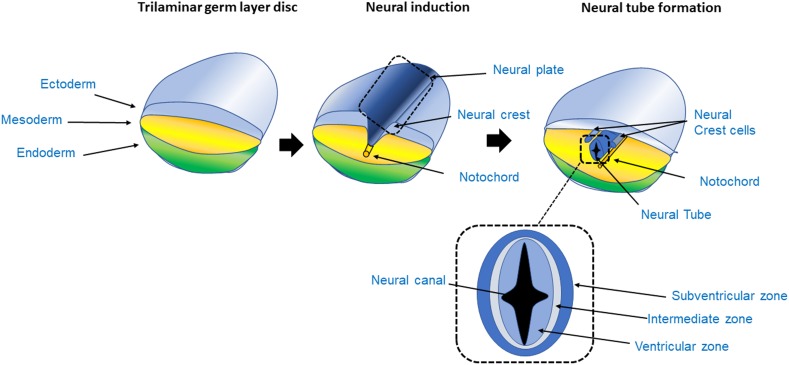
Process of neurogenesis. The CNS originates from the ectoderm layer of the trilaminar germ disc. The process of neurogenesis begins with the formation of the neuroepithelium from the neuroectoderm, giving rise to the neural tube in a process called primary neurulation (Johns, 2014). The trilaminar germ layer disc is composed of the three germ layer tissues, the endoderm, mesoderm and ectoderm. The mesoderm gives rise to the notochord, a tubular mesodermal structure which on releasing trophic factors, triggers neural induction, whereby uncommitted or naïve ectoderm becomes committed to the neural lineage, and subsequently stimulates the formation of the neural tube in the overlying ectoderm (Dickinson et al., 1995). As the ectoderm acquires a neuroectoderm identity, it forms a fold, initially giving rise to the neural plate and subsequently forms the neural fold. The grooves at either side of the fold are called the neural crest. The crest then detaches from the margins of the neural fold giving rise to the peripheral nervous system. The neural plate continues to fold on itself giving rise to the hollow neural tube; the lumen of the neural tube is called neural canal. As the neural tube closes, it forms a fluid filled cavity that generates the ventricular zone, an area occupied by progenitor cells such as neuroblasts and glioblasts (Johns, 2014; Brodal, 2016; Kelava and Lancaster, 2016a). In the transverse section of the neural tube the VZ, intermediate zone and subventricular area can be seen. In the VZ mitosis takes place generating radial glia during neurogenesis.

The study of neurogenesis (summarised in Figure 1), neural processes and neurological disorders is a very challenging science, as the brain is a uniquely complex organ and is largely inaccessible for experimental investigations in living humans, which is mostly limited to discarded post-surgical tissue samples or neuroimaging, transcranial magnetic stimulation and electroencephalography studies (Komssi and Kähkönen, 2006; Stan et al., 2006; Brammer, 2009; Eyal et al., 2016).

While animal models have appreciably advanced the understanding of human brain development and neurodegenerative diseases, the inherent developmental, anatomical and physiological differences between the central nervous system (CNS) of animals and the human can add complexity to the interpretation of findings (Elston et al., 2001; DeFelipe et al., 2002; Roth and Dicke, 2005; Herculano-Houzel, 2009; Mohan et al., 2015). A detailed discussion of these models would go beyond the scope of this review and it has been reported elsewhere (Chesselet and Carmichael, 2012; Dawson et al., 2018). As such, the current understanding of human brain development has been limited to common features shared with other animal species (Kelava and Lancaster, 2016b). Although centuries of human *post mortem* tissue examinations have contributed to the fundaments of modern neuroscience, allowing the study of specific features of the human brain, these tissues cannot be implemented in functional studies (Filis et al., 2010; Kelava and Lancaster, 2016a). Consequently, researchers have strived to develop and optimise *in vitro* neural culture systems for advancing the understanding of the functioning of the CNS and the underlying pathogenesis of neurological diseases. Animal models, *ex vivo* and *post mortem* tissues have been utilised in other areas of brain research.

The seminal work of the pioneering “fathers” of neuroscience and Nobel laureates, Santiago Ramón y Cajal and Camillo Golgi provided the foundations for investigating the intricacies of the human nervous system’s macro and micro anatomy (Ramón y Cajal, 1904; Golgi, 1906). In his published volumes, Santiago Ramón y Cajal artistically summarised his work describing the structure and organisation of the vertebrate nervous systems and discussed his theories including, amongst others, the “neuron doctrine,” the law of dynamic, functional or axipetal polarisation of electrical activity in neurons and his ideas on neurogenesis, neural plasticity and neuronal regeneration/degeneration (Ramón y Cajal, 1894, 1904, 1909, 1913). Since then, neuroscientists have strived on the wealth of knowledge inherited from Cajal and Golgi, who immensely contributed to the evolution of modern neuroscience over these centuries.

**FIGURE 2:**
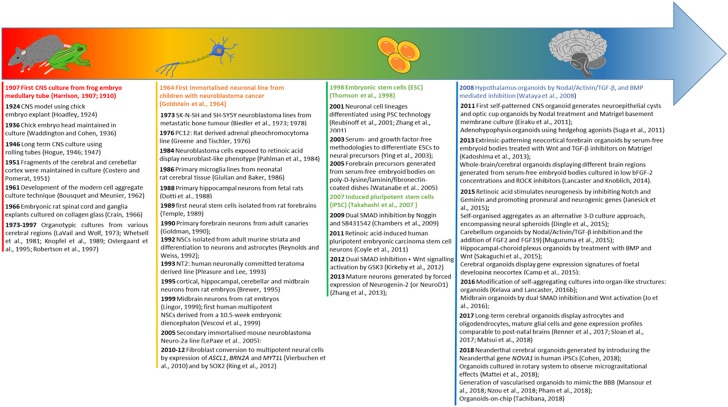
Evolution timeline of CNS modelling. The timeline illustrates the evolution from organ explants to the use of 2-D neural cell lines, and subsequently a shift toward pluripotent stem cell derived neural cultures leading to the development of CNS specific organoids. For each category of modelling a time excursus is presented chronologically over 100 years.

In this review, we present an evolutionary overview of CNS modelling through an historical excursus (Figure 2), starting from the origins of neural cell cultures from tissue explants and organotypic cultures, to cell monolayers, aggregates and ultimately leading to the generation of complex three-dimensional (3-D) cultures such as cerebral organoids from patient-specific isolated cells, emphasising the growing excitement for the latter in the quest for the most representative human CNS model.

## Tissue Explants and Organotypic Cultures

The first *in vitro* nervous system culture was established by Ross Harrison in 1907, where frog embryo grafts consisting of pieces of medullary tubes were cultured as hanging drops in lymph. Although Harrison was able to observe neurite extensions and maintained the culture for up to 4 weeks, it was not possible to generate permanent specimens with intact nerve fibres (Harrison, 1907, 1910). Decades later, the first culture of intact CNS from chick embryos was established, permitting the recapitulation of the developing brain architecture *in vitro*, by displaying the formation of early retinal tissue (Hoadley, 1924; Waddington and Cohen, 1936).

The original long term culture (up to 143 days) of human foetal brains was established in 1946 by Mary Jane Hogue by using the roller tube approach (Hogue, 1946, 1947). Costero and Pomerat (1951) successfully cultured neurons obtained from the cerebral and cerebellar cortex explants of adult human brains, for up to 5 weeks, using Maximow’s flying-drop.

The first CNS organotypic culture was pioneered by Bousquet and Meunier (1962) using rat hypophysis. In 1966, Crain cultured explants from embryonic rat spinal cord and ganglia on collagen coated glass demonstrating that grafted neural tissue possessed organotypic differentiation and bioelectric properties for electrophysiological studies (Crain, 1966). Since then, brain slices of several cerebral areas have been established as organotypic cultures, including the hippocampus, substantia nigra, locus coeruleus, striatum and basal forebrain (LaVail and Wolf, 1973; Whetsell et al., 1981; Knopfel et al., 1989; Ostergaard et al., 1995; Robertson et al., 1997).

Although tissue explants and organotypic slice cultures more accurately recapitulate the cerebral cytoarchitecture, they are difficult to acquire and cell specific functional studies are subject to severe limitations (Kelava and Lancaster, 2016b). For instance, the handling of organotypic preparations remains quite challenging with respect to preserving the sterility, viability and the cytoarchitecture of the tissues (Walsh et al., 2005). Additionally, cell maturation in culture may differ within the explanted tissues, with some cell types displaying a mature phenotype while others remain immature, being dependent on the age of the subject at the time of tissue collection (Gähwiler, 1981).

## 2-D Neural Cell Cultures

The improvement in the ability to maintain cell cultures for extended periods has enabled a range of isolated primary nerve cell cultures to be established, including hippocampal neurons derived from rat foetuses (Dotti et al., 1988), cortical, hippocampal, cerebellar and midbrain neurons from rat embryos (Brewer, 1995; Lingor et al., 1999), forebrain neurons of adult canaries (Goldman, 1990) and primary microglia from cerebral tissues of neonatal rats (Giulian and Baker, 1986). The generation of glial cell cultures, viable for several weeks, was also achieved as described in the seminal study by McCarthy and De Vellis; dissociated cerebral cortices of 1–2 days old rat pups brains were used to isolate primary astrocytes and oligodendrocytes that were devoid of any viable neuronal cell (McCarthy and de Vellis, 1980).

Culturing of primary neural cells, however, is hampered by a limited culture lifespan and the finite number of achievable passages with non-proliferating quiescent mature neurons (Gordon et al., 2013). To overcome this, the first neural stem cells (NSCs) were isolated from rat forebrains in 1989, establishing a self-renewing line of multipotent progenitors with the plasticity to generate progenies of the main neuronal phenotypes (Temple, 1989).

In 1992, Reynold and Weiss demonstrated the presence of NSCs in the adult CNS of murine brains through the isolation of nestin expressing cells from the striata and inducing their differentiation into neurons and astrocytes *in vitro*, thereby establishing appropriate culture conditions to demonstrate the functional attributes of these stem cells (Reynolds and Weiss, 1992). The availability of NSCs facilitated the culture of neuronal or glial cells, without the need for complex and laborious isolations of the latter cells from whole explants (Gordon et al., 2013).

In parallel, the development of immortalised cell lines eliminated the need for multiple acquisitions of tissue for neural cell culturing. The first immortalised neuronal line was derived from lymph nodes, infiltrated bone marrow and liver tissue of children with neuroblastoma cancer; these cells were cultured *in vitro* for up to 1 year and were capable of differentiating into tissues resembling mature ganglion cells (Goldstein et al., 1964). However due to the clinical heterogeneity of neuroblastoma, cultured cells were characterised by morphological variability, and thus efforts were made to develop more defined cell lines and improve the longevity of cultures (Biedler et al., 1973). This led to the generation of the SK-N-SH neuroblastoma cell line from metastatic bone tumour (Biedler et al., 1973), which was further subcloned to establish the widely used SH-SY5Y neuroblastoma line (Biedler et al., 1978).

To induce cells to display a more neuronal phenotype, the culture environment can be manipulated by the addition of growth factors and signalling molecules such as retinoids and dibutyryl cAMP (Kuff and Fewell, 1980; Kovalevich and Langford, 2013); this is exemplified by the experiment conducted by Pahlman et al. (1984), where neuroblastoma cells were exposed to retinoic acid to display a neuroblast-like phenotype expressing immature neuronal markers (Pahlman et al., 1984).

Other secondary immortalised cell lines developed for modelling neuronal cells include the mouse neuroblastoma Neuro-2a (LePage et al., 2005), PC12, a rat derived adrenal pheochromocytoma line (Greene and Tischler, 1976), the immortalised LUHMES cell line from human embryonic mesencephalic tissue and NT2 cells, a human neuronally committed teratoma derived line capable of differentiating into a mixed population of neuronal and glial cells under retinoic acid exposure (Pleasure and Lee, 1993; Coyle et al., 2011).

In neurobiology, the majority of primary neuronal tissue cultures is derived from animal sources, and as such, the techniques used to develop them suffered the same limitations of animal models, such as costs, ethical considerations, the obvious inter-species differences and the incorrect assumption that orthologous genes share similar functions in closely related living systems (Hartung, 2008; Gharib and Robinson-Rechavi, 2011; Ko and Frampton, 2016; Shipley et al., 2016). Moreover, the main concern with using immortalised cell lines for the study of neurobiology and for modelling neurological conditions, is that these cells contain genetic and metabolic abnormalities which may not represent a normal cell or those of human patients (Gordon et al., 2013; Carter and Shieh, 2015).

In 1999, Vescovi et al. established the first human multipotent NSCs derived from a 10.5-week embryonic diencephalon (Vescovi et al., 1999). The establishment of human NSCs opened exciting opportunities in neurobiology, since normal cells of human derivation, with self-renewing and long-term culturing capabilities, could be used for the generation of a multitude of functional neuronal and glial progenies for disease modelling and potential clinical applications (Carpenter et al., 1999; Jakel et al., 2004).

Although efforts for the successful long-term *in vitro* culturing of NSCs have been made (Sun et al., 2008), these cells were found to be incapable of accurately representing stem cells *in vivo*, due to their inability to recapitulate the entire range of neural lineages and hence brain development (Conti and Cattaneo, 2010; Kelava and Lancaster, 2016b).

More recently, multipotent neural cells were obtained by direct conversion of fibroblasts by the ectopic expression of *ASCL1*, *BRN2A* and *MYT1L* (Vierbuchen et al., 2010) or by the sole expression of *SOX2* (Ring et al., 2012). However, it is not clear to what extent the reprogrammed neural progenitors are capable of retaining epigenetic memory and the fidelity of the resemblance with neural progenitors is yet to be determined (Velasco et al., 2014).

## Human Pluripotent Stem Cell-Derived Neural Cultures

**FIGURE 3:**
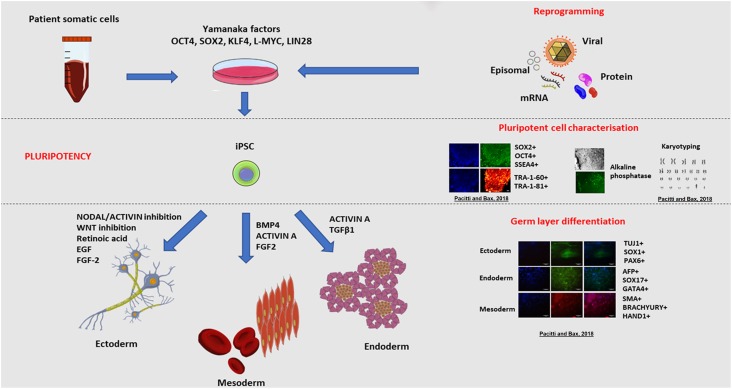
Induced pluripotent stem cell (iPSC) derivation and characterisation. The ectopic overexpression of the transcription factors *OCT3/4*, *KLF4*, *SOX2*, *L-MYC*, *LIN28*, known to be expressed in ESCs, reverts mature somatic cells such as dermal fibroblasts and peripheral blood mononuclear cells (PBMC) to display a pluripotent embryonic-like phenotype (Takahashi et al., 2007; Okita et al., 2013). Transfection of cells with vectors expressing these transcription factors enables the epigenetic reprogramming of cells, through a series of stochastic events, to express endogenous OCT4 and NANOG, the characteristic transcription factors determining the pluripotency, self-renewal and proliferative capacity of cells (Lohle et al., 2012). In synergy, the ectopic overexpression of these genes triggers a sequence of epigenetic modifications leading to DNA demethylation and chromatin changes that eventually result in the acquisition of a pluripotent state in transfected cells (Jaenisch and Young, 2008). A multitude of vectors have been used to deliver the reprogramming factors and these approaches are broadly divided into non-viral and viral, and integration and non-integration methods. For instance, reprogramming could be achieved using viral vectors including retroviruses, lentiviruses and more recently Sendai non-integrating virus. Alternatively, non-viral methods include mRNA or protein delivery or transient expression achieved with episomal plasmids. Pluripotent stem cells are defined by the presence of specific markers including cell surface proteoglycans (TRA-1-60 and TRA-1-81) and glycosphingolipids (SSEA-3 and SSEA-4) and the expression of transcription factors OCT4 and SOX2 (Thomson et al., 1998; Tonge et al., 2011). The resulting pluripotent cells, have the same embryonic plasticity for differentiating into almost any tissue type of the three germ layers (endoderm, mesoderm and ectoderm) when stimulated by the appropriate signalling molecules and growth factors (Itskovitz-Eldor et al., 2000; Okita et al., 2013). Examples of cells derived from the germ layers include nervous and epidermal tissue from the ectoderm, haematopoietic and muscle cells from the mesoderm, and pancreatic cells from the endoderm. Copyright permission was obtained for the reproduction of images taken from Pacitti and Bax (2018).

The advent of human embryonic stem cells (ESCs) in 1998 (Thomson et al., 1998) and then human induced pluripotent stem cells (iPSC) in 2007 (Takahashi et al., 2007), have provided exciting prospects in the field of neuroscience. The tremendous plasticity of these cells as an unlimited source of specific cell types, and their replicative capacity *in vitro*, rendered them the ideal candidate for neurodevelopmental studies. In particular, the possibility to generate neuronal cells directly from iPS cells derived from patients affected by a specific disorder provides an unprecedented opportunity to study the very phenotype of these diseases *in vitro*. Figure 3, summarises the different methods of derivation of iPSCs and the various characterisation criteria for qualifying as pluripotent cells.

The differentiation of ESCs *in vitro* reproduces with great fidelity the *in vivo* neuroectoderm formation (Wu et al., 2010), and indeed, neuronal cells were amongst the first lineages to be differentiated using PSC technology (Reubinoff et al., 2001; Zhang et al., 2001). This was first achieved using ESCs, by inducing their neuronal differentiation in spheroid-like aggregates of cells [called embryoid bodies (EBs)], cultured in serum-free conditions to selectively promote the growth of neural cells, which self-organised to form rosettes (Zhang et al., 2001). These rosettes generated structures reminiscent of neural tubes (Curchoe et al., 2012), organised as progenitor zones resembling the ventricular and subventricular zones (SVZs) with the presence of radial glia (Shi et al., 2012b; Edri et al., 2015).

Subsequent studies improved methodologies to differentiate ESCs to neural precursors in the complete absence of serum or growth factors (Ying et al., 2003). The combination of the embryoid body-derived rosette and the serum free media provided the foundation for the serum-free embryoid bodies culture, which in the presence of inductive signals, including Wnt and Nodal antagonists (Dkk1 and LeftyA, respectively) and Sonic hedgehog could generate forebrain (telencephalic) precursors on poly-D-lysine/laminin/fibronectin coated dishes (Watanabe et al., 2005) and could be further differentiated into cortical neurons (Gaspard et al., 2008).

However, because ESCs are of embryonic origin, they are subjected to considerable ethical and practical issues. The development of iPSCs has since introduced an advantageous tool for the study of neurodevelopment and neuropathology; the possibility of generating ESC-like cells from adult somatic cells, not only circumvents issues related to ethics and sample acquisition, but also provides the advantage of developing pluripotent lines directly from diseased patients, and hence the study of neurobiology and neurological disorders accounting for genetic variations within a more heterogeneous cohort of relevant genotypes/phenotypes (Avior et al., 2016). In fact, the same differentiation protocols can be applied to the generation of iPSCs for the provision of neural progenitors and specific neural lineages (Mariani et al., 2012).

Several different PSC differentiation methods were developed, although these are highly variable and unpredictable due to undefined factors such as the use of neural inducing stromal feeder cells, the heterogeneous nature of embryoid bodies and inefficient methods for the selective survival of neural cells (Schwartz et al., 2008; Denham and Dottori, 2011).

The development of neuralization protocols for PSCs has been one of the main areas of investigation in neuroscience and it is being achieved by the improved understanding of the underlying signalling pathways involved, leading to the development of more efficient methodologies such as dual SMAD inhibition (Chambers et al., 2009). The dual SMAD inhibition was accomplished by using Noggin and the small molecule SB431542 to inhibit the NODAL/Activin, TGF-β and bone morphogenic protein (BMP) signalling, thereby inhibiting the differentiation into cells with a non-neural fate (Chambers et al., 2009; Pauklin and Vallier, 2015). The dual SMAD inhibition method obviated the need for stromal cells and embryoid body based techniques and permitted the efficient generation of a broad repertoire of PSC-derived neural progenitors within shorter differentiation times in adherent monolayer cultures (Chambers et al., 2009). The dual SMAD inhibition method was further improved by the addition of a glycogen synthase kinase 3 (GSK3) inhibitor to induce Wnt signalling activation, yielding progenitors which matched the gene expression profiles of developing foetal brains, and with a broad range of regional differentiation phenotypes, from rostro-caudal to midbrain and dorso-ventral patterning of neural progenitors (Kirkeby et al., 2012). In another experiment, dual SMAD inhibition was combined with retinoid signalling to enhance differentiation of PSCs to cortical neurons (Shi et al., 2012b).

Lineage priming of PSCs to the neural lineage has also been achieved by the forced expression of the single transcription factor Neurogenin-2 (or NeuroD1) which yielded the generation of mature neurons expressing glutamatergic receptors and forming spontaneous synaptic networks within 2 weeks from transfection (Zhang et al., 2013). Another study demonstrated that the forced synergistic expression of the transcription factors ASCL1 and DLX2 induced the differentiation of PSC to near pure GABAergic neurons (Yang et al., 2017).

Another common differentiation protocol uses retinoic acid treatment on embryoid bodies for promoting commitment to the neural lineage (Schuldiner et al., 2001). In participation with FGF and Wnt, retinoic acid is a potent caudalizing factor of the neuroectoderm and is a differentiation-inducing molecule essential for the development of the neural crest and the generation of cortical neurons (Villanueva et al., 2002; Diez del Corral and Storey, 2004; Siegenthaler et al., 2009). Retinoic acid has been demonstrated to inhibit neural proliferation and promote neurogenesis by inhibiting the expression of genes that negatively regulate neuronal differentiation (such as Notch and Geminin), while promoting the expression of proneural and neurogenic genes (Janesick et al., 2015). However, it is important to note that although retinoids play a fundamental regulatory role during neural tube formation, their function in neural development and axial patterning are strictly context, time and dose-dependent, and therefore it is crucial to include retinoids at an appropriate differentiation stage in cultures (Maden, 2002).

## Monolayer Cultures to Organoids

With the improvement of differentiation methods, culturing techniques have also been refined to introduce structural complexities that better recapitulate the *in vivo* development and cytoarchitecture. Availability of this technology has led to a tremendous interest from researchers worldwide, particularly from the prospective of generating more representative models of the human phenotype, and as a means to “replace, reduce and refine” the use of animal models (Sneddon et al., 2017). The culture substrate is as essential to neural cell culture as the use of appropriate inducing factors. In the CNS, extra cellular matrixes (ECMs) are crucial for cell migration and differentiation, therefore *in vitro* substrates are fundamental for the preferred differentiation of NSCs and support of differentiated cells (Franco and Müller, 2011).

The best-described substrates used for the *in vitro* culture of neural cells (whether NSC or PSC-derived) are poly-L-ornithine, poly-L-lysine, fibronectin, collagen and laminin (Ge et al., 2015). All these substrates have been found to support neural differentiation to differing degrees (Ma et al., 2008). One study reports that poly-L-ornithine induces preferred differentiation of NSCs into neurons and oligodendrocytes, compared to poly-L-lysine and fibronectin (Ge et al., 2015). However, other studies suggested that laminin or laminin-rich substrates enhance differentiation of NSC to neurons (Hall et al., 2008; Ma et al., 2008).

Nevertheless, the ECM is a complex mixture of molecules (laminins, proteoglycans, collagens) and therefore specific combinations of substrates may be necessary to better reproduce the *in vivo* scenario (Ma et al., 2008; Franco and Müller, 2011). Hydrogel scaffolds such as Matrigel, which consist of a mixture of extracellular molecules including laminin, collagen IV, heparan sulphate proteoglycans and entactin (Kleinman and Martin, 2005) are often successfully employed for the culture and long-term support of neural cells (Ma et al., 2008; Lee et al., 2015). Matrigel is not only advantageous as a substrate for monolayer cultures, but due to its polymerizing nature, it can be utilised as a semi-solid scaffold matrix for 3-D cultures (Tibbitt and Anseth, 2009). However, even Matrigel is not immune from major limitations, being an animal-derived matrix characterised by batch variation and with an undefined composition that may produce a source of variability in experimental conditions (Hughes et al., 2010).

Traditional 2-dimensional (2-D) culturing has contributed immensely to the understanding of neuroscience but is substantially limited for recapitulating the *in vivo* complexities of the CNS. Advancement in cell culturing techniques for the generation of neural cell lines has greatly improved with the directed differentiation of iPSCs into monolayers of specific neural cell types (Pasca et al., 2014; Barral and Kurian, 2016; McKinney, 2017). A summary of various CNS neural tissues derived from the differentiation of iPSC is presented in Table 1.

**Table 1:** Summary of different CNS neural cells differentiated from iPSC.

Disease group	Disease modelled	Genetic/chromosomal abnormality	iPSC-derived cells	Reprogramming method	Reference
Lysosomal storage disorders	Jansky–Bielschowsky disease	*CLN5*	CLN5 neurons	Sendai-virus transduction	Uusi-Rauva et al., 2017
	Batten disease	*CLN3, TPP1*	CLN3 neurons	Retrovirus transduction	Lojewski et al., 2014
	Pompe disease	*GAA*	Pompe neurons	Retrovirus transduction	Higuchi et al., 2014
	Niemann-Pick type C1	*NPC1*	NPC1 neurons, astrocytes	Retrovirus transduction, lentivirus transduction	[Bibr B247][Bibr B59]
Metabolic disorders	Lesch–Nyhan syndrome	*HPRT*	Neurons	Retrovirus transduction	Mastrangelo et al., 2012
	Gaucher’s disease	*GBA1*	Dopaminergic neurons, neurons	Lentivirus transduction	[Bibr B183][Bibr B245][Bibr B7][Bibr B234]
	Metachromatic leukodystrophy	*ARSA*	Neural stem cells, astroglial progenitor cells	Retrovirus transduction	Doerr et al., 2015
	X-linked Adrenoleukodystrophy	*ABCD1*	Neurons, astrocytes, oligodendrocytes	Retrovirus transduction, lentivirus transduction	[Bibr B113][Bibr B8]
Neurodegenerative disorders	Parkinson’s disease (PD), idiopathic PD	*LRRK2,?*	Midbrain dopaminergic neurons	Cre-recombinase excisable viruses, retrovirus transduction	[Bibr B229][Bibr B175][Bibr B214][Bibr B66]
	Amyotrophic lateral sclerosis (ALS)	*TDP-43,SOD1*	Spinal motor neurons, astrocytes	Retrovirus transduction, TALEN transfection, sendai-virus transduction, episomal transfection	[Bibr B15][Bibr B60][Bibr B220][Bibr B274][Bibr B12]
Neurodevelopmental disorders	Rett syndrome	*TRPC6, MECP2*	Neural progenitor cells, glutamatergic neurons, astrocytes	Retrovirus transduction	[Bibr B172][Bibr B154][Bibr B4][Bibr B121][Bibr B264][Bibr B53][Bibr B88][Bibr B239]
	Atypical Rett syndrome	*CDKL5*	Glutamatergic neurons, GABAergic neurons	Lentivirus transduction, retrovirus transduction	[Bibr B3][Bibr B208]
	Timothy syndrome	*CACNA1C*	Neural progenitor cells, cortical glutamatergic neurons	Retrovirus transduction	[Bibr B188][Bibr B243]
	Down Syndrome	Trisomy 21	Cortical glutamatergic neurons	Lentivirus transduction	Shi et al., 2012a
	Familial dysautonomia	*IKBKAP*	Neural crest precursors	Lentivirus transduction	Lee et al., 2009, 2012
	Fragile X Syndrome	*FMR1*	Neural progenitor cells, forebrain neurons, glial cells	Retrovirus transduction, episomal transfection	[Bibr B250][Bibr B55][Bibr B184][Bibr B89]
	Cockayne syndrome	*ERCC6*	Neural progenitor cells	Sendai-virus transduction	Vessoni et al., 2016
	Angelman/Prader-Willi syndromes	*UBE3A*	Neurons, astrocytes	Retrovirus transduction	Chamberlain et al., 2010
	Phelan-McDermid syndrome	22q13 deletion	Forebrain neurons	Retrovirus transduction	Shcheglovitov et al., 2013
Neuropsychiatric diseases	Frontotemporal dementia (FTD)	*CHMP2B, C9ORF72*	Forebrain cortical neurons	Retrovirus transduction, episomal transfection	[Bibr B2][Bibr B281]
Epilepsy	Dravet syndrome	*SCN1A*	Dopaminergic, GABAergic, glutamatergic neurons, forebrain interneurons, glial cells	Retrovirus transduction	[Bibr B100][Bibr B115][Bibr B148], 2016
	Early infantile epileptic encephalopathy	*STXBP1*	GABAergic, glutamatergic neurons	Episomal transfection	Yamashita et al., 2016
Neuromuscular disorders	Spinal muscular atrophy (SMA)	*SMN1*	Forebrain, sensory, motor neurons, astrocytes	Lentivirus transduction, retrovirus transduction, episomal transfection	[Bibr B57][Bibr B31][Bibr B215][Bibr B36][Bibr B161][Bibr B217][Bibr B278][Bibr B19][Bibr B174][Bibr B176][Bibr B145][Bibr B72][Bibr B269][Bibr B189]
Movement disorders	Huntington’s disease	*HTT*	Medium spiny neurons	Lentivirus transduction	The Hd iPsc Consortium, 2012
	Hereditary spastic paraplegia	*SPG3A, SPG4, SPG11, ATL1, SPAST*	Cortical neural progenitor cells, forebrain, glutamatergic neurons	Lentivirus transduction, episomal transfection, retrovirus transduction	[Bibr B47][Bibr B96][Bibr B282][Bibr B167]
	Ataxia telangiectasia	*ATM*	Neural progenitor cells, GABAergic neurons	Lentivirus transduction	Nayler et al., 2012 Carlessi et al., 2014
	Friedrich’s ataxia	*FXN*	Neural progenitor cells, neural crest cells, peripheral sensory neurons, glial cells	Retrovirus transduction, lentivirus transduction, transposon transfection	[Bibr B147][Bibr B61][Bibr B98][Bibr B16]


*Neural cells characteristic of the CNS have been generated by directed differentiation of pluripotent stem cell derived lines, to recapitulate specific cell types or disease condition*.

However, these 2-D cultures are unlikely to recapitulate the intricate cytoarchitecture, the elaborate network of diverse neural cell types and the functional complexity of the *in vivo* CNS (Paşca, 2018). Cell differentiation and maturation is critically dependent on both intrinsic and extrinsic cues originating from the interactions with various neural cells and ECM molecules, and the cross-talk and dynamic interaction of neural cells is crucial for the recapitulation of a physiologically relevant system (Gomes et al., 2001; Rowitch and Kriegstein, 2010; Jiang and Nardelli, 2016).

Nevertheless, 2-D cultures have the advantage of a greater scalability, with less complex directed differentiation approaches and facilitated imaging. However, 2-D cells also favour stronger interactions with the surfaces of the culturing vessel rather than cell–cell interactions or between cells and the ECM, altering their proliferation and differentiation capabilities (Paşca, 2018). Three dimensional models can overcome 2-D culturing limitations, allowing the dynamic interactions between cells and ECMs and the recreation of signalling, metabolites and oxygen gradients across the culture (Ko and Frampton, 2016).

A variety of 3-D culture methods have been developed, with one example in the form of tissue explant cultures, such as whole brain sections grown in culture dishes or microfluidic devices (Ullrich et al., 2011; Huang et al., 2012).

For instance, recently, an organotypic human Alzheimer disease model consisting of a 3-D triculture system of neurons, astrocytes and microglia co-cultured in a microfluidic system was engineered. Authors developed this 3-D culture system to model the neuroinflammation and neurodegeneration aspects of the disease, employing human iPSC-derived NSCs overexpressing mutant *APP* and *PSEN1* genes associated with familial Alzheimer’s. This system successfully recapitulated the tauopathy, β-amyloid accumulation and microglia mediated neuroinflammation more efficiently than the 2-D models (Park et al., 2018).

However, these cultures are often problematic to maintain in the long-term and are generally derived from animal sources, and hence not representative of human development (Ko and Frampton, 2016). Self-organised aggregate cultures are an alternative 3-D *in vitro* culture approach, encompassing neural spheroids (Dingle et al., 2015). Further sophistication of culture methods has led to the modification of self-aggregating cultures for the generation of organ-like structures, termed organoids (Kelava and Lancaster, 2016b).

**FIGURE 4:**
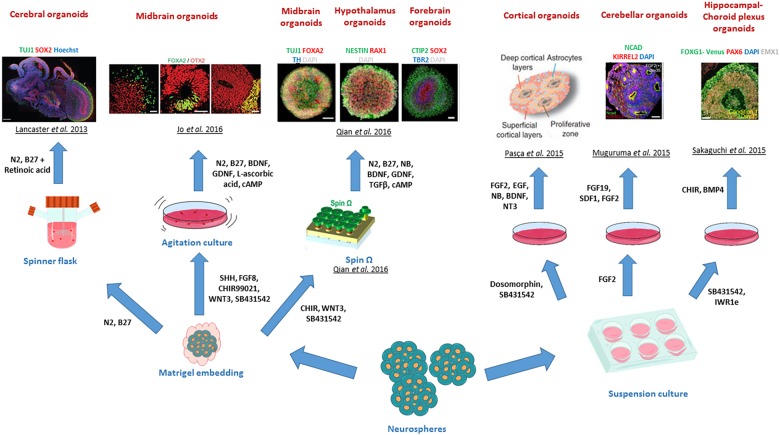
Different brain regional identities recapitulated by CNS organoids. The diagram summarises the various patterning methods developed to generate region specific CNS organoids. Copyright permission was obtained for the reproduction of images taken from Lancaster et al. (2013); Muguruma et al. (2015), Pasca et al. (2015); Sakaguchi et al. (2015), Jo et al. (2016); Qian et al. (2016).

Central nervous system organoids can be divided in two categories based on their patterning approach, either being self-patterned or extrinsically patterned. Self-patterned organoids are organoids that are cultured without the addition of exogenous morphogens that favour specific brain regions, whereas extrinsically patterned organoids refer to the generation of brain specific regional identities via the addition of morphogens and neurotrophic factors such as FGF, Sonic Hedgehog, Nodal, Wnt and BMP (Clevers, 2016; Brawner et al., 2017). Figure 4 illustrates the different brain regionals identities recapitulated in CNS organoids.

To illustrate this, the seminal study of Eiraku et al. (2008) demonstrated that serum-free embryoid bodies with quick reaggregation are capable of self-organising into stratified cortical tissues in cultures, thus showing that ESCs spontaneously differentiate toward a neural state by default. This notion provided the fundaments to generate the first self-patterned CNS organoid, employing serum-free embryoid bodies to generate neuroepithelial cysts, which further self-organised into optic cup organoids, to present regions with retinal identities upon treatment with Nodal protein and culture on Matrigel basement membrane (Eiraku et al., 2011).

Extrinsic-patterning organoids can be exemplified by the generation of the first dorsal telencephalon organoid recapitulating a human cerebral cortex at 8–10-week gestation by Mariani et al. (2012), where serum-free embryoid bodies were treated with Wnt and TGF-β inhibitors and cultured on poly-L-ornithine, laminin and fibronectin for up to 70 days. Similarly, other adaptations of the method developed by Eiraku and colleagues led to the generation of neocortical forebrain organoids maintained on Matrigel for over 3 months (Kadoshima et al., 2013). Further modifications of protocols for the generation of organoids patterned with other brain specific regions and sub-regions have been developed to model the adenohypophysis (using hedgehog agonists) (Suga et al., 2011), hypothalamus (by Nodal/Activin/TGF-β, and BMP mediated inhibition) (Wataya et al., 2008), cerebellum (using the Nodal/Activin/TGF-β inhibition and the addition of FGF2 and FGF19) (Muguruma et al., 2015), midbrain (by using the dual SMAD inhibition and Wnt activation) (Jo et al., 2016) and hippocampal-choroid plexus (by treatment with BMP and Wnt) (Sakaguchi et al., 2015). Combining the strong neuralizing dual SMAD inhibition with the serum-free embryoid body approach also enabled the generation of cortical spheroids, which represented 3-D tissues containing neurons and astrocytes and mimicked cortical development stages up to the mid-foetal period (Pasca et al., 2015).

**FIGURE 5:**
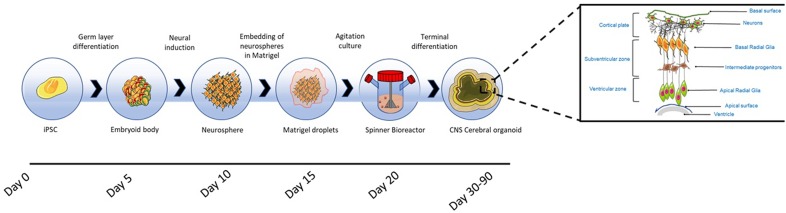
Cerebral organoid formation. Cerebral organoids originate from pluripotent stem cell derived embryoid bodies cultured in low concentrations of bFGF-2 and with ROCK inhibitors to prevent anoikis. The embryoid bodies are differentiated toward neuroectoderm, resulting in the formation of neurospheres, which are embedded into Matrigel hydrogel droplets. After the embedding, budding neuroepithelia generate fluid filled cavities reminiscent of ventricles. The neuroepithelium begins to migrate outwardly to generate the cortical layers consisting of Cajal-Retzius cells, deep and superficial cortical progenitors. On exposure to retinoic acid, cerebral organoids self-organise through self-patterning mechanisms to display diverse populations of neural progenitors including radial glia, which expand forming cerebral structures (Lancaster and Knoblich, 2014). The region in which radial glia originate, recapitulates the *in vivo* VZ and SVZ (Kelava and Lancaster, 2016a). Prior to neurogenesis, radial glia are formed from the neuroepithelial cells and facilitate the migration of the neural and glial progenitors (Howard et al., 2008; Johns, 2014); these cells are characterised by the asymmetric self-renewal division leading to the generation of one neuron and one radial glia (Gotz and Huttner, 2005; Lancaster and Knoblich, 2012). Radial glia migrate, establishing the SVZ, where cells divide symmetrically forming intermediate progenitors (Haubensak et al., 2004; Miyata et al., 2004). Radial glia produce outer radial glia in the SVZ, which are self-renewing (Fietz et al., 2010; Hansen et al., 2010). Radial glia residing in the VZ divide at the apical surface adjacent to the ventricle. Intermediate progenitors and basal radial glia migrate basally to the SVZ. Neurons formed from radial glia, migrate radially from the ventricular and SVZ toward the basal region to establish the cortical plate (Johns, 2014; Kelava and Lancaster, 2016a). The neuronal migration depends on a layer of Cajal-Retzius cells and the outward migration is regulated by the protein reelin secreted by the latter cells (Frotscher, 1998). Within the intermediate zone of the cerebral hemispheres, neurons undergo apoptosis, and the region becomes the subcortical white matter (Johns, 2014).

Lancaster and Knoblich (2014) introduced, for the first time, the concept of whole-brain organoid or cerebral organoids (Figure 5), representing different brain regions within the same 3-D platform. Cerebral organoids, are generated from PSC-derived serum-free embryoid bodies obtained by culturing in low concentrations of bFGF-2 and with ROCK inhibitors to promote cell survival (Lancaster and Knoblich, 2014). The serum-free embryoid bodies are subsequently induced to differentiate to the neuroectoderm within suspension cultures, establishing a radially organised neuroepithelium around the spheroid (Lancaster and Knoblich, 2014; Sutcliffe and Lancaster, 2017).

The resulting neurospheres are embedded into Matrigel hydrogel droplets, allowing the growth of continuous and orientated neuroepithelial buds and, the apicobasal expansion of the layer of neuroepithelial cells throughout the ECM basement scaffold. After the embedding, budding neuroepithelia generate fluid filled cavities reminiscent of ventricles. The neuroepithelium begins to migrate outwardly to generate the cortical layers consisting of Cajal-Retzius cells, deep and superficial cortical progenitors. On exposure to retinoic acid, cerebral organoids self-organise through self-patterning mechanisms to display sparse populations of neural progenitors including radial glia, which begin to expand forming cerebral structures (Lancaster and Knoblich, 2014). Retinoic acid is only added for terminal differentiation, as being a potent caudalizing factor it inhibits neurogenesis in the early stages of neuroepithelium formation (Petros et al., 2011).

The region in which radial glia originate, recapitulates the *in vivo* ventricular zone (VZ) and SVZ (Kelava and Lancaster, 2016a). Therefore, similar to *in vivo* neurogenesis, neural progenitors migrate to form the cortical plate, spontaneously giving rise to distinct brain regions reminiscent of the dorsal cortex, ventral forebrain, hindbrain, midbrain, retina, hippocampus and choroid plexus (Lancaster and Knoblich, 2014).

As cerebral organoids expand in culture, the neuronal population increases in number, resulting in the enlargement of tissues, reaching sizes of up to 4 mm in diameter (Sutcliffe and Lancaster, 2017). The whole development process requires 7–10 days for the generation of neurospheres, and more than 20 days before the appearance of the first mature neural population (Lancaster and Knoblich, 2014; Sutcliffe and Lancaster, 2017). After 1 month in culture the cerebral tissue begins to thicken, showing tissues of different regional identities, as evidenced by the expression of FOXG1 (forebrain), TTR (choroid plexus), FZD9 (hippocampus) and SOX2 (VZ radial glia) (Lancaster and Knoblich, 2014). Later studies have confirmed that cerebral organoids display with high fidelity the gene expression signatures of a foetal developing neocortex (Camp et al., 2015).

The increase in size introduces problems with regard to nutrient and oxygen diffusion through the central regions of tissue, causing necrosis (Lancaster and Knoblich, 2014). Therefore, the organoids are grown in spinning bioreactors to provide agitation and maximise oxygen and nutrient exchange (Kelava and Lancaster, 2016b). Within bioreactors, cerebral organoids are capable of displaying a longevity of up to 1 year, although it has been reported that growth becomes stationary after 5 months, with organoids shrinking in size in subsequent months due to neuronal loss and disappearance of progenitors (Lancaster and Knoblich, 2014).

## Modelling Neurological Disorders With Cns Organoids

**FIGURE 6:**
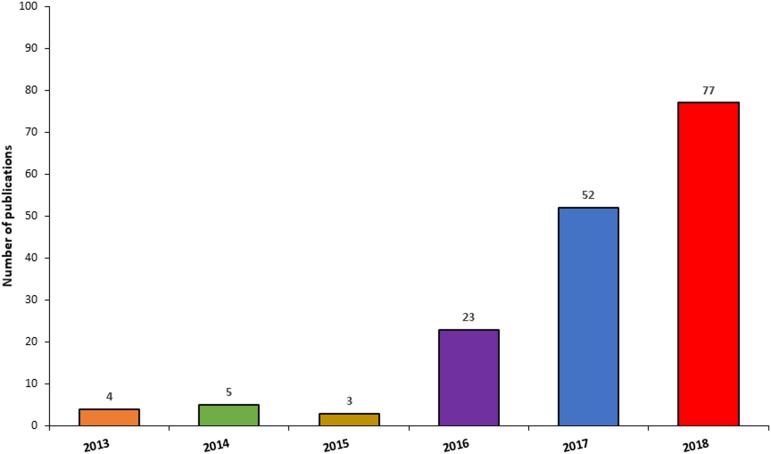
Frequency of publications reporting the use of CNS organoids by year. The chart illustrates a surge in publications involving the use of CNS organoids, between 2014 and 2018.

Since the generation of the first brain organoids, unsurprisingly, there has been an exponential surge in publications employing this technology (Figure 6), due to their amenability for the study of neurodevelopment and neurological diseases, but also as a potential platform for the development of novel neurotherapeutics. When searching Pubmed using the terms “Cerebral organoid,” “Brain organoid,” “CNS organoid,” and “Cortical organoid” the number of publications for the year 2013 were 4, compared to 52 and 77 for the years 2017 and 2018, respectively. Due to the significant expansion and importance of this technology, this section of the review will primarily focus on the applications and advancements in this field. Cerebral organoids are indeed a relatively new platform but are finding wider application as *in vitro* disease modelling tools, not only for many developmental disorders, but also for psychiatric diseases and neurodegenerative conditions (Lancaster et al., 2013; Mariani et al., 2015; Garcez et al., 2016; Jo et al., 2016; Kelava and Lancaster, 2016a; Raja et al., 2016; Bershteyn et al., 2017; Birey et al., 2017; Iefremova et al., 2017). The advent of genome editing techniques such as CRISPR/Cas9 has changed the scene for tackling genetic disorders and opened a new chapter for potential stem cell applications in the clinic (Waddington et al., 2016). In particular, organoids’ versatility and adaptability to genome editing techniques or gene therapy approaches make them valuable candidates for the identification and testing of novel therapeutic approaches (Yin et al., 2016; Gonzalez-Cordero et al., 2018).

### Microcephaly

Lancaster and colleagues generated organoids from a patient harbouring a *CDK5RAP2* mutation, and demonstrated that the organoids from the patient with microcephaly were smaller compared to a healthy control. This was explained by fewer proliferating progenitors and a premature differentiation in the disease tissues. The group also demonstrated that the phenotype could be rescued by inducing the expression of the wild-type gene (Lancaster et al., 2013).

Furthermore, cerebral organoids have aided the understanding of microcephaly associated with neuroinfection with the Zika virus. In the study of Garcez et al. (2016) human cortical organoids infected with Zika virus exhibited a 40% decrease in size compared to non-infected controls. An elegant study by Qian et al. (2016) introduced an innovation in culturing cerebral organoids through the use of miniaturised spinning bioreactors (the SpinΩ), and the authors determined that infection of organoids with Zika virus resulted in an increase in apoptosis with consequent reduction in cell proliferation and a marked thinning of the ventricular zone. Another study found that human iPSC-derived cerebral organoids infected with Zika virus had a decrease in PAX6 expressing progenitors in the proliferative zones and consequently a decrease in differentiated neurons leading to a disruption in the cortical plate formation (Cugola et al., 2016). Dang et al. (2016) found that Zika virus infection in organoids induced a significant upregulation of Toll-like receptor 3 gene, leading to apoptosis and dysregulation of neurogenesis.

### Macrocephaly

Li et al. (2017) demonstrated the recapitulation of macrocephaly in cerebral organoids, by the genetic ablation of *PTEN* using CRISPR/Cas9. Edited organoids exhibited an activation in AKT signalling in neural progenitors regulating cortical maturation, resulting in a delay in neuronal differentiation, an increase in cellular proliferation or radial glia progenitors, and a significant increment in organoid sizes (Li et al., 2017).

### Autistic Spectrum Disorder

Mariani and colleagues employed iPSCs derived from patients with idiopathic autism spectrum disorder to generate cerebral organoids; the patient tissues exhibited an overgrowth of neurites and synapses, in a similar fashion to that observed in post mortem brain investigations of individuals with autism. The same study observed an increased production of GABA-ergic inhibitory interneurons in patient organoids, while also identifying an upregulation of *FOXG1*, thus indicating a correlation between the two observations (Mariani et al., 2015). Using cerebral organoids combined with monolayer cultures, Mellios et al. (2017) characterised defects in neurogenesis associated with MeCP2 deficiency in Rett syndrome, which consisted primarily in expanded ventricular zones with enhanced PAX6+ ventricle-like structures. In this study, through the inhibition of miR-199, affected signalling pathways (PKB/AKT and ERG/MAPK) were rescued and the dysregulations in neural differentiation ameliorated (Mellios et al., 2017).

### Miller-Dieker Syndrome

Miller-Dieker syndrome, a form of lissencephaly, was modelled using patient-derived iPSCs, enabling the identification of a stalled mitosis of outer radial glia, thus suggesting that dysfunctions in progenitor cell cycles could be a feature of cortical malformations in the disease (Bershteyn et al., 2017). Another study identified non-cell autonomous defects in Wnt signalling associated with Miller-Diekers syndrome (Iefremova et al., 2017).

### Sandhoff Disease

Allende et al. (2018) developed cerebral organoids from iPSCs derived from an infant with Sandhoff disease and from isogenic controls corrected for the *HEXB* mutation by gene editing. The authors demonstrated that GM2 ganglioside only accumulated in the disease organoids and affected organoids displayed an increase in size and cellular proliferation compared to the isogenic control counterpart (Allende et al., 2018).

### Schizophrenia

In their study examining the association of *DISC1* gene with schizophrenia, Ye et al. (2017) used human cortical organoids to demonstrate that DISC1/Ndel1 interactions regulate mitotic events in NSCs and that a delay in mitosis was observed in organoids derived from a patient with a *DISC1* mutation.

### Modelling Prenatal and Perinatal Drug Exposure

Cerebral organoids have been used to investigate how the prenatal exposure to substances of abuse including illegal drugs, alcohol and tobacco affect neurogenesis. A study examining the exposure of cocaine, demonstrated that cytochrome P450 CYP3A5-mediated oxidative metabolism was responsible for the developmental abnormalities of the foetal neocortex, resulting in the inhibition of neuroepithelial progenitor proliferation, premature neuronal differentiation and a reduction in the cortical plate formation (Lee et al., 2017). Zhu et al. (2017a) investigated the effects of ethanol exposure in organoids to better understand alcohol-induced defects in neurogenesis in foetal alcohol syndrome, and where able to identify through a transcriptome analysis, gene expression alterations in the Hippo pathway and in other genes including *GSX2* and *RSPO2*. Another study using organoids-on-chip investigated the effect of nicotine on neural development, demonstrating a disruption in cortical development in exposed organoids (Wang et al., 2018). The use of organoids for studies on the effect of drug exposure on embryonic CNS development would not be limited to substances of abuse, but could also be used in the context of neurodevelopmental toxicity whereby toxicological profiles of compounds on teratogenicity or neurotoxicity could also be assessed in the system, such as for the evaluation of the neural teratogenic effect of valproic acid or the effect of environmental chemicals (Schwartz et al., 2015; Miranda et al., 2018b; Wood et al., 2018).

### Alzheimer’s Disease

The limitations of cerebral organoids in recapitulating only early neurogenesis, hinders their application for studying late-onset neurodegenerative diseases such as Alzheimer’s, Parkinson’s and Huntington’s (Kelava and Lancaster, 2016a). Nevertheless, Raja et al. (2016) developed cerebral organoids derived from patients with early onset familial Alzheimer’s, and were able to recapitulate the disease phenotype through the demonstration of β-amyloid aggregation, hyperphosphorylated tau proteins and abnormal endosomes. The authors further demonstrated that β-amyloid and tau pathologies were significantly reduced when treating patient organoids with β and γ secretase inhibitors (Raja et al., 2016).

## Other Developments and Applications

More recently, research groups have sought to optimise and advance methods for the generation of more reproducible and morphologically complex organoids. For instance, Basuodan et al. (2018) have generated 3-D cultures with characteristics similar to cerebral organoids by transplanting iPSC-derived neurospheres embedded in ECM matrix, into brains of ischaemic mice (Basuodan et al., 2018).

Organoids also provide a powerful tool for the study of evolutionary developmental biology, and for comparing neurogenesis between species *in vitro* (Giandomenico and Lancaster, 2017). More recently, due to the advancement in gene editing technologies, such as CRISPR/Cas9, Neanderthal cerebral organoids were generated by introducing the Neanderthal gene *NOVA1* in human iPSCs. This study demonstrated that Neanderthal brain organoids resembled to a certain degree, organoids generated from patients with autism, indicating that these similarities may be linked to socialisation behaviours (Cohen, 2018).

Moreover, organoids have been used to study cellular migration, cross-talk and circuitry assembly by either generating region-specific organoids and fusing them, or by co-culturing cells from different lineages in a single organoid, and thus generating structures referred to as assembloids (Birey et al., 2017; Workman et al., 2017; Pham et al., 2018). By using this approach, forebrain assembloids derived from patients with Timothy syndrome, were shown to have defects in the migration of cortical interneurons, and these could be restored pharmacologically by modulating the mutated L-type calcium channel, thus contributing significantly to the understanding of epilepsy and autism associated with the syndrome (Birey et al., 2017).

The substantial versatility of organoid applications is demonstrated in the interesting experiment conducted by Mattei and colleagues, where cerebral organoids were employed to investigate how neurogenesis and neural development could be affected by microgravity. In projection of spaceflight advancements, the authors cultured human organoids in rotary cell culture system to demonstrate that microgravitational changes influenced the expression of rostral-caudal patterning genes and cortical markers (Mattei et al., 2018).

Literature reports on the ability of organoids to recapitulate the composition of an adult CNS were elusive in the initial phases. Seminal studies on the comparison of cerebral organoids to foetal brains, initially suggested that the development of fully matured cerebral organoids could only parallel the early embryonic cerebral development observed at 8–10 weeks gestation (Mariani et al., 2012; Kelava and Lancaster, 2016a,b). However single cell sequencing experiments have revealed that organoids are capable of replicating late-mid foetal periods of a 19–24 weeks gestational brain (Pasca et al., 2015).

Given the ability of organoids to recapitulate developmental timing, much interest has also been directed at the types of CNS neural cell populations which compose organoids and their maturation stage, such as glial cells. A recent study conducted by Monzel et al. (2017) reported the presence of differentiated glial cells in midbrain organoids from as early as day 27 of development, with myelinating oligodendrocytes ensheathing neurites at day 61 (Monzel et al., 2017). Moreover, the same study demonstrated that after 61 days, mature astrocytes staining for S100β and AQP4 characterised mature midbrain organoids (Monzel et al., 2017). Additional studies have further demonstrated that cerebral organoids cultured for prolonged periods, ranging from months up to 1.5 years, displayed the presence of differentiated astrocytes and oligodendrocytes (Camp et al., 2015; Renner et al., 2017; Matsui et al., 2018). Furthermore, using culture times of over a year has been shown to yield organoids exhibiting a large proportion of mature glial cells and gene expression profiles comparable to those of post-natal brains (Renner et al., 2017; Sloan et al., 2017).

Our group has used cerebral organoids to model mitochondrial neurogastrointestinal encephalomyopathy (MNGIE), a rare metabolic disorder which manifests with leukoencephalopathy amongst other neurological and gastrointestinal symptoms (Pacitti, 2018; Pacitti and Bax, 2018). The ability of organoids to produce differentiated astrocytes and myelinating oligodendrocytes, and most importantly the recreation of a physiologically relevant cross-talk between cells has been a great asset for investigating the leukodystrophic manifestations of the disease and shed light on the poorly understood pathomolecular mechanisms of the CNS involvement in MNGIE (Pacitti, 2018; Pacitti and Bax, 2018).

In comparison to 2-D cultures, brain organoids represent a valuable tool for the study of glial cells as, for instance, when dealing with astrocytes, traditional monolayer cultures are inadequate since the morphological complexity and the vast heterogeneity cannot be appropriately modelled (Imura et al., 2006; Lange et al., 2012; Puschmann et al., 2013). Also, astrocytes cultured in 2-D preferentially interact with plastic surfaces rather than between cells and the ECM (Paşca, 2018). Most importantly, astrocytes in 2-D cultures tend to have an undesirably high baseline reactivity, possibly caused by serum components, although this can be minimised by using serum-free neurobasal formulations (Foo et al., 2011; Pekny and Pekna, 2014; Pasca et al., 2015). However, iPSC-derived astroglial cells in 2-D cultures require extensive timing to allow for maturation, and thus practicalities inherent to long term culturing of cell monolayers, for appropriate astrocyte maturation, represent a major challenge (Dezonne et al., 2017; Sloan et al., 2017). In addition to cell intrinsic properties, astrocyte maturation may require interactions with other neural cells types, which would not be represented in pure astrocyte cultures differentiated by pluripotent cells, unless specifically co-cultured after differentiation (Chandrasekaran et al., 2016).

Three dimensional cultures, like cerebral organoids, allow the recreation of a more physiological spatial environment that favours a representative organisation of astrocytes and their interactions with other neural cells and ECM components (Pasca et al., 2015; Liddelow and Barres, 2017). Compared to 2-D cultures, 3-D cultures have indeed demonstrated a better capacity for recapitulating astrocyte heterogeneity (Puschmann et al., 2013; Puschmann et al., 2014; Liddelow and Barres, 2017). Moreover, in 3-D cultures, basal reactivity of astrocytes is negligible, rendering them the ideal platform for the study of the heterogeneous spectrum of astrocyte subtypes and their activation (Puschmann et al., 2013; Pasca et al., 2015; Liddelow and Barres, 2017).

## Current Caveats and Advancement in the Organoid Technology

While cerebral organoids offer an advantageous culture system with diversified neural cells for modelling as closely as possible the intercellular interactions during organogenesis, the technology also suffers from some limitations, which are constantly being addressed with ongoing research efforts. One of the greatest limitations of the 3-D platform is the confounding batch variability (Di Lullo and Kriegstein, 2017; Paşca, 2018). Cell differentiation relies on spontaneous events that are characterised by a high degree of stochasticity as they lack developmental axes (Paşca, 2018). This results in regional identities that could differ in distribution, composition and densities between organoids, generating concerns regarding reproducibility, accuracy and scalability (Di Lullo and Kriegstein, 2017). The spontaneous self-patterning mechanisms on which cerebral organoids rely, results in the formation of several brain regional identities, when compared to brain region-specific organoids. As such, cerebral organoids are characterised by a great level of heterogeneity and complexity, which result in morphological variabilities between and within organoid batches, leading to inherent reproducibility issues (Lancaster et al., 2013; Kelava and Lancaster, 2016a). Referring to this elevated variability, Lancaster and colleagues suggested that if using organoids to detect phenotypes in the context of genetic disorders, defects must be robust enough to be noticeable (Lancaster and Knoblich, 2014; Kelava and Lancaster, 2016b; Giandomenico and Lancaster, 2017). In fact, organoid variability could have severe implications with respect to disease modelling, drug screening or neurodevelopmental studies, as the heterogeneity could affect the consistency of phenotypes exhibited, masking true differences between diseased and healthy, or treated and non-treated tissues. Organoid variability would appear to be partly accountable to a bioreactor-based effect, meaning that a more controlled growth microenvironment would contribute to a better reproducibility (Quadrato et al., 2017).

More recently, polymer microfilaments were implemented as scaffolds to promote a more elongated generation of embryoid bodies, which has been found to enhance neuroectoderm formation and cortical development, and also reduce the issues of reproducibility and variability observed in the regional identities of filament scaffolded organoids (Lancaster et al., 2017).

Contrarily to the “intrinsic” self-patterning protocol, patterning of organoids using inductive signals and optimised bioreactors, as conducted by Qian et al. (2016), led to the development of more consistent region specific organoids which were less influenced by batch variability. Optimal patterning and the relevant reproduction of proper developmental axes requires a spatiotemporally defined gradients of morphogens, which is challenging to achieve in culture; it has been suggested that a way to circumvent this could be through the use of slow-releasing microbeads to establish a morphogen gradient (Lee et al., 2011; Sun et al., 2018). In contrast, a recent study revealed that the removal of inductive factors such as those used for the dual SMAD inhibition during the EB differentiation stage, or refraining from using maturating growth factors in culture medium during the organoid stage (such as BDNF, GDNF and TGF-β), yields more optimal organoids with reduced inter and intra batch variability in terms of reproducibility, size, growth and neural cell composition and maturity (Yakoub and Sadek, 2018). Cerebral organoids generated through this optimised protocol exhibited a robust neuronal zone and positive staining for general neuronal and mature astrocytic markers, and were characterised by a strong upregulation of neurotransmitter receptor genes involved in synaptic functions including the glutamate, α-amino-3-hydroxy-5-methyl-4-isoxazolepropionic acid (AMPA) receptor GluA1, and the *N*-methyl-D-aspartate (NMDA) receptors GluN1, GluN2A and GluN2B, and the γ-amino butyric acid (GABA) receptor GABA-B receptor 1 (Yakoub, 2019).

Potentially, the elevated variability observed in EB preparations may contribute to the heterogeneity observed between organoid preparations (Wilson et al., 2014). Therefore, controlling this heterogeneity, deriving from the spatial disorganisation and asynchronous differentiation of EB aggregates, could further minimise reproducibility issues observed during organoid development (Miranda et al., 2015, 2018a). The use of centrifugal forced-aggregation and silicon micro-textured surfaces improved symmetry, size and synchronised differentiation in EB, increasing consistency between preparations (Ungrin et al., 2008). Another example of a possible bioengineering solution to control aggregate size and size by cellular confinement, could be identified in the use of microfabrication technologies where organoids cultured on a micropillar array exhibited robust brain regionalization and cortical organisation (Zhu et al., 2017b).

Organoids lack some cells of the CNS including endothelial cells composing the cerebral vasculature, the blood–brain barrier (BBB), and microglia, as these do not derive from ectodermal tissues (Di Lullo and Kriegstein, 2017). These cells are found to have a role in CNS development via extrinsic signals that induce maturation and differentiation of neural cells including astrocyte and cortical neurons (Stubbs et al., 2009; Cunningham et al., 2013; Sloan et al., 2017).

The lack of vascularisation has been reported to prevent the delivery of oxygen and small molecules deep inside the tissue, often resulting in necrosis within the centre of the organoids. Most importantly, the lack of vascularisation interferes with certain patterning cues necessary for organoid development and progenitor differentiation. Late developing brains are highly dependent on vascularisation as niches of neural progenitors, such as the SVZ, are generally found in proximity of vessels. The solution to the limitations inherent to vascularisation and stochastic patterning cues can only be sought by refinement of the existing protocols, by either modifying culture conditions to mimic the physiological environment as closely as possible or through bio-engineering innovation to provide a flowing system of nutrients to organoids to reproduce vascularisation (Kelava and Lancaster, 2016a).

Recently, it has been suggested that combining organoid culture with microfluidic technology may circumvent the vascularisation issue, for example, by culturing endothelial cells in microfluidic channels (Auger et al., 2013) to provide a flow system of nutrients and trophic molecules, thus allowing the *in vitro* modelling of organoid angiogenesis (Yin et al., 2016). Having highlighted the lack of vascularisation and BBB as major limitations of cerebral organoids, it has been envisaged that the introduction of further structural complexities may enhance the spectrum of applications of this platform (Kelava and Lancaster, 2016a). Several groups have addressed the lack of the BBB by generating vascularised organoids (Mansour et al., 2018; Nzou et al., 2018; Pham et al., 2018). Pham et al. (2018) generated vascularised cerebral organoids by re-embedding organoids in Matrigel droplets, seeded with iPSC-derived endothelial cells. Mansour et al. (2018), employed a different approach and achieved the vascularisation of human organoids, through engraftment in murine cortices *in vivo*. This demonstrated the feasibility of integration with the host, an improvement in viability and longevity of the tissue, a synaptic connectivity of transplanted organoids and the host, and ultimately, the formation of a microvascular network in the grafted organoids (Mansour et al., 2018). Also, Nzou et al. (2018) generated a six cell type cortical organoid consisting of astrocytes, pericytes, oligodendrocytes, NSCs and vascular endothelial cells, creating a functional BBB expressing tight and adherent junctions to examine barrier permeability using neurotoxic compounds.

The structural complexity of cerebral organoids has its pros and cons. Whereas the high degree of neural cell diversity and complex cross-talks are an advantage, this may also represent a disadvantage when trying to test hypotheses related to the contribution of individual cell types to mechanistic processes. The complementation of a 3-D model with a 2-D cell culture system of purified cells of interest from the organoids would allow the compartmentalisation and investigation of individual neural cell types, enabling molecular mechanisms intrinsic to specific cell types to be teased out.

## Concluding Remarks

Studies of neural development and neurodegenerative diseases present many challenges due to the structural and functional complexity of the CNS, together with the limited possibility of *in vivo* experimental manipulation. Although animal models have contributed to the current knowledge, there are significant structural, cellular and molecular differences in the CNS of animal and humans, making data extrapolation and interpretation a formidable task. The past 100 years have seen the evolution of a number of culture systems for modelling the human CNS. Tissue explants and organotypic cultures were replaced by 2-D cultures thereby permitting investigation in more controlled systems. Issues of tissue availability were addressed by the development of human neural cell lines derived from tumours and more recently, the discovery of NSCs has permitted the generation of neuronal and glial cells in large quantities. Three-dimensional culture systems (organoids) are the most recent technological development in CNS modelling and bridge the gap between native tissue and 2-D cell cultures. Many advancements have been made in CNS organoid development, as evidenced by the ability of culturing for prolonged times, the potential to recapitulate late brain developmental milestones and *in vivo* transplantation. However, ethical and epistemological issues have been raised around organoids questioning their potential for developing consciousness (Lavazza and Massimini, 2018; Shepherd, 2018). At present, organoids can only recapitulate early stages of development and can be used in a relatively narrow spectrum of applications. However, their use is currently not free of hindrances and thus continuous efforts must be made for further improvement to overcome their limitations for a more appropriate and reliable use. One of the major improvements can be found in organoids-on-chip, which as opposed to traditional organoids, are not self-assembled but are rather constructed to produce a more reliable and consistent culture, through the inclusion of engineered elements such as biosensors and microfluidic channels (Tachibana, 2018). At present, the excitement for this technology is driving elegant research worldwide and it holds the potential for promising and revolutionary applications.

## Author Contributions

DP, RP, and BB contributed to the conception, writing, and review of the manuscript.

## Conflict of Interest Statement

The authors declare that the research was conducted in the absence of any commercial or financial relationships that could be construed as a potential conflict of interest.
